# Explainable Convolutional Neural Networks for Brain Cancer Detection and Localisation

**DOI:** 10.3390/s23177614

**Published:** 2023-09-02

**Authors:** Francesco Mercaldo, Luca Brunese, Fabio Martinelli, Antonella Santone, Mario Cesarelli

**Affiliations:** 1 Department of Medicine and Health Sciences “Vincenzo Tiberio”, University of Molise, 86100 Campobasso, Italy; luca.brunese@unimol.it (L.B.); antonella.santone@unimol.it (A.S.); 2 Institute for Informatics and Telematics, National Research Council of Italy, 56121 Pisa, Italy; 3 Department of Engineering, University of Sannio, 82100 Benevento, Italy; mcesarelli@unisannio.it

**Keywords:** brain, machine learning, deep learning, explainability

## Abstract

Brain cancer is widely recognised as one of the most aggressive types of tumors. In fact, approximately 70% of patients diagnosed with this malignant cancer do not survive. In this paper, we propose a method aimed to detect and localise brain cancer, starting from the analysis of magnetic resonance images. The proposed method exploits deep learning, in particular convolutional neural networks and class activation mapping, in order to provide explainability by highlighting the areas of the medical image related to brain cancer (from the model point of view). We evaluate the proposed method with 3000 magnetic resonances using a free available dataset. The results we obtained are encouraging. We reach an accuracy ranging from 97.83% to 99.67% in brain cancer detection by exploiting four different models: VGG16, ResNet50, Alex_Net, and MobileNet, thus showing the effectiveness of the proposed method.

## 1. Introduction

Brain cancer is characterised by the growth of abnormal cells or a cluster of cells in the brain or its surrounding structures. Brain tumors, a type of brain cancer, can be categorised as either malignant (cancerous) or benign (non-cancerous). Malignant brain tumors have the ability to invade nearby tissues and metastasise to other parts of the body, whereas benign tumors typically do not invade nearby tissues or spread. Brain tumors are a significant cause of cancer-related fatalities in children under the age of 20. In fact, brain tumors have surpassed acute lymphoblastic leukemia as the primary cause of solid cancer deaths in this particular age group. This emphasises the importance of understanding and addressing brain tumors in pediatric oncology (http://blog.braintumor.org/, accessed on 20 August 2023). Brain tumors are, indeed, a significant cause of solid cancer deaths among young adults between the ages of 20 and 39. They rank as the third leading cause of solid cancer-related deaths in this age group. Each year, more than 5000 individuals lose their lives to brain tumors. Furthermore, in the United Kingdom, it is estimated that at least 102,000 children and adults are currently living with a brain tumor. This statistic underscores the prevalence and impact of brain tumors on individuals and their families (https://www.cancerresearchuk.org/health-professional/cancer-statistics/statistics-by-cancer-type/brain-other-cns-and-intracranial-tumours, accessed on 20 August 2023). Brain tumors can, indeed, have a significant impact on life expectancy. On average, individuals diagnosed with brain tumors may experience a reduction in life expectancy by around 20 years. This is considered one of the highest reductions in life expectancy among all types of cancer. Survival rates for brain tumors can vary depending on various factors such as the type of tumor, its grade, location, and individual characteristics. It is important to note that the survival rate mentioned, stating that only 19% of adults survive for five years after a cancer diagnosis, is a general statistic and may not apply uniformly to all types and stages of brain tumors. Survival rates can vary widely and are influenced by many factors (https://www.cancer.net/cancer-types/brain-tumor/statistics, accessed on 20 August 2023).

Brain cancers can, indeed, impact physical and cognitive abilities due to their location in the control center for movement and emotion. The brain is responsible for coordinating various bodily functions, including movement, sensory perception, cognition, and emotional regulation. When brain tumors develop in these critical areas, they can disrupt the normal functioning of these processes.

The specific effects on physical and cognitive abilities will depend on the size, location, and growth pattern of the tumor, as well as individual factors. Some common symptoms of brain tumors can include headaches, seizures, changes in motor skills, difficulty with speech and language, memory problems, and emotional changes.

Treatment approaches, such as surgery, radiation therapy, and chemotherapy, aim to target and manage brain tumors while minimising damage to the surrounding healthy tissues. Rehabilitation therapies, such as physical therapy, occupational therapy, and speech therapy, may also be employed to help patients regain or adapt to changes in their physical and cognitive abilities.

Astrocytomas are, indeed, the most common type of primary brain tumor among the group known as gliomas, with about 15,000 new astrocytomas diagnosed every year in the United States. Males are slightly more affected than females, with a ratio of 1.3/1 (https://www.aans.org/Patients/Neurosurgical-Conditions-and-Treatments/Astrocytoma-Tumors, accessed on 20 August 2023) Astrocytomas are a type of brain tumor that originates from astrocytes, a type of glial cell in the brain. They are classified based on their grade, which indicates their level of aggressiveness and malignancy.

The exact cause or motivation behind the development of astrocytomas is not fully understood. Like other types of cancer, astrocytomas are thought to result from a combination of genetic and environmental factors. Certain genetic mutations and abnormalities have been associated with the development of astrocytomas, but the specific triggers or underlying causes are still being investigated (https://www.abta.org/tumor_types/astrocytoma/, accessed on 20 August 2023).

The World Health Organisation (WHO) has a classification system for brain tumors, including astrocytomas, which is based on four grades. They are crucial for maintaining and managing information processing in the brain. The grading system helps to determine the severity and aggressiveness of the tumor, as well as guide treatment decisions. The grades for astrocytomas range from I to IV, with each grade indicating a different level of malignancy:

1. Grade I: Pilocytic astrocytoma. These are the least aggressive astrocytomas. They are often referred to as pilocytic astrocytomas and typically have well-defined borders. These are typically slow-growing and have a good prognosis.
2. Grade II: Diffuse astrocytoma. These are low-grade astrocytomas. They show some abnormal characteristics in the tumor cells and may have infiltrative growth into nearby tissues. These are low-grade tumors with a moderate potential to become more aggressive over time.
3. Grade III: Anaplastic astrocytoma. These are anaplastic astrocytomas. They are considered intermediate-grade tumors with more abnormal cells and more aggressive behavior compared to grade II tumors. These are high-grade tumors that tend to grow more quickly and are more aggressive than Grade II astrocytomas.
4. Grade IV: Glioblastoma (also known as glioblastoma multiforme). These are the most malignant and aggressive astrocytomas known as glioblastomas. Glioblastomas are high-grade tumors characterised by highly abnormal and rapidly dividing cells. They are invasive and have a tendency to infiltrate surrounding brain tissue. This is the most aggressive and malignant type of astrocytoma with a poor prognosis.

The WHO grading system helps healthcare professionals in determining the appropriate treatment strategy and predicting the likely behavior and outcome of astrocytomas based on their grade (https://braintumor.org/wp-content/assets/WHO-Re-Classification-2016_FINAL.pdf, accessed on 20 August 2023). Astrocytomas can be categorised into low-grade and high-grade types. Low-grade astrocytomas are typically localised and exhibit slow growth, while high-grade astrocytomas grow rapidly and necessitate a distinct treatment approach. Among astrocytoma tumors in children, the majority are low-grade, while in adults, most of them are high-grade.

Anaplastic astrocytoma belongs to Grade III in the astrocytoma classification. These tumors originate from astrocytes, a type of star-shaped brain cell that forms part of the glial tissue surrounding and protecting nerve cells in the brain and spinal cord. Gliomas are a group of tumors that arise from glial tissue, which includes astrocytomas. The symptoms of anaplastic astrocytomas differ based on the tumor’s size and location within the brain or spinal cord.

Astrocytoma Grade IV, also known as glioblastoma, is a highly aggressive type of cancer characterised by a significant portion of tumor cells continuously reproducing and dividing. Glioblastomas are invasive and have the ability to infiltrate neighboring regions of the brain and, in some cases, they can even spread to the opposite side of the brain through connecting fibers. Common symptoms of glioblastomas include headaches, personality changes, nausea, and stroke-like symptoms, which can progress to unconsciousness. Due to their rapid spread into other parts of the brain, glioblastomas are considered the most aggressive and infiltrative form of brain cancer (https://www.thebraintumourcharity.org/understanding-brain-tumours/types-of-brain-tumour-adult/glioblastoma/, accessed on 20 August 2023) and they represent the most common malignant brain tumor in adults.

The accurate and timely diagnosis of brain cancer at an early stage is, indeed, crucial for patient care and the planning of future treatment. Early diagnosis allows healthcare professionals to initiate appropriate interventions promptly, potentially improving treatment outcomes and quality of life for patients.

The early detection of brain cancer enables medical professionals to:

- Determine the exact type and grade of the brain tumor: A precise diagnosis helps guide treatment decisions and allows healthcare teams to tailor therapies specifically to the patient’s condition.
- Plan the most effective treatment approach: Based on the diagnosis, doctors can develop a comprehensive treatment plan, which may include surgery, radiation therapy, chemotherapy, targeted therapies, or a combination of these approaches.
- Manage symptoms and improve patient comfort: Early diagnosis facilitates the early management of symptoms associated with brain cancer, such as headaches, seizures, cognitive changes, and motor difficulties. This can help improve the patient’s overall well-being and quality of life.
- Monitor disease progression and response to treatment: With an early diagnosis, healthcare professionals can closely monitor the tumor’s progression and assess the response to treatment. This allows for timely adjustments to the treatment plan if needed.

In this context, deep learning [1,2,3,4,5,6,7] can be very helpful in reaching a diagnosis. For this reason, in this paper, we propose a method aimed at discriminating between medical images related to brain cancer and healthy patients. The proposed method, by analysing a brain magnetic resonance image, is able to detect the presence of brain cancer. Furthermore, the proposed approach is able to highlight the areas of the brain image symptomatic of cancer and, for this reason, the proposed method is devoted to localising the disease areas, thus providing the explainability [8] behind the classifier decision. For this task, we consider the Gradient-Weighted Class Activation Mapping (Grad-CAM) [8] algorithm. As a matter of fact, by exploiting the proposed method in the real-world, the doctor is not only able to obtain, in an automatic way, the prediction of the brain cancer disease but he/she is able to see the areas that have been responsible for that particular decision by the model. We think this aspect can provide confidence for medical personnel in the real-world adoption of automatic techniques for disease classification and localisation.

In the following, we itemise the main contributions of the paper:

- A method aimed at detecting medical images related to brain cancer, by means of explainabile deep learning, is proposed;
- Four different deep learning networks are exploited, i.e., VGG16, Resnet50, Alex_Net, and MobileNet;
- We provide prediction explainability by considering the Grad-CAM, aimed at localising the area on the medical images responsible for the brain cancer detection, thus providing a valuable tool for radiologists and domain experts;
- A dataset composed of 600 patients is analysed, obtaining an accuracy equal to 99.67%;
- A comparison (in terms of the number of analysed patients, accuracy, the focus of the paper, and localisation) between the proposed method and the state-of-the-art is proposed with the aim of better highlighting the effectiveness of our method.

## 2. Literature Survey

This section provides an overview of the latest advancements in brain cancer detection utilising machine learning methods.

Ramteke et al. [9] conducted a study exploring the statistical texture features extracted from both normal and malignant MRIs. They utilised the Nearest Neighbors classifier as their classification algorithm and achieved an accuracy rate of 80% in their classification task.

Isselmou and colleagues [10] present an approach for distinguishing between benign and malignant brain tumors through the analysis of MRI data. Their method achieves an accuracy of approximately 95% in this discrimination task.

Sharma and colleagues, in reference [11], investigated the use of Multilayer Perceptron (MLP) and Naive Bayes classification algorithms for distinguishing between malignant and benign brain tumors based on texture features. They employed a 66% percentage split for training, and the remaining instances were used for testing. The results showed a maximum accuracy of 98.6% for MLP and 91.6% for Naive Bayes in the detection of malignant and benign brain cancer using a dataset of 212 brain MRIs.

Babu and Varadarajan [12] explore the efficacy of gray level co-occurrence features in discerning between malignant and benign brain cancer MRIs using the Support Vector Machine classification algorithm.

Gadpayle and colleagues [13] investigate models that leverage texture features and employ Neural Network and Nearest Neighbors classifiers to categorise brain MRIs into normal or abnormal brain conditions. They achieve an accuracy of 70% with the Nearest Neighbors classifier and 72.5% with the Neural Network classifier.

Jafari and colleagues, in reference [14], present a hybrid approach that combines Genetic Algorithm and Support Vector Machine for brain cancer detection. The features used in this approach include statistical, wavelet, and frequency transformation features. The average accuracy achieved by the method is 83.22%, with results ranging between 79% and 87%.

Chaddad et al. [15] suggest the adoption of Gaussian mixture model features to discriminate between benign brain MRIs and those affected by Glioblastoma in their study.

Kharrat et al., in reference [16], utilise a feature set based on 2D Wavelet Transform and Spatial Gray Level Dependence Matrix to distinguish between 83 brain-cancer-affected and healthy patients. They employ the Support Vector Machine supervised machine learning algorithm for this discrimination task, similarly to researchers in references [12,14].

Ghosh and Bandyopadhyay [17] utilise the Fuzzy C-Means clustering algorithm to determine whether the MRI area under analysis in 45 patients is associated with brain cancer or not.

Zahran et al. [18] explore a neural-network-based approach using two-dimensional discrete wavelet transform features. They find that their network demonstrates superior classification performance for normal MRIs compared to malignant ones, achieving an overall accuracy of 0.83.

In reference [19], Khawaldeh et al. develop a five-layer convolutional neural network (CNN) to classify brain MRIs as either healthy or unhealthy. Specifically, the unhealthy brain tumor images are further categorised into low and high grades, resulting in an impressive accuracy of 0.91.

In reference [20], Zacharaki et al. investigate a machine learning model that utilises both texture and shape features to distinguish between low- and high-grade brain cancer MRIs. The authors employ the Support Vector Machine algorithm to generate the model for this classification task.

El-dahshan et al. [21] introduce a framework for detecting malignant brain cancer MRIs and benign ones. They utilise features based on discrete wavelet transform and apply principal component analysis to reduce the feature vector’s dimensionality. The classification of MRIs is performed using a forward back-propagation neural network.

In reference [22], the authors employ a multi-layer feedforward neural network with automated Bayesian regularisation to classify brain tumor MRIs and non-brain tumor MRIs. Their method is evaluated using a dataset of nine pediatric patients.

In the work presented in [23], El and colleagues consider the features derived from discrete wavelet transformations to construct two classifiers. The first one is based on a feedforward back-propagation artificial neural network, while the second one relies on k-nearest neighbor. These classifiers aim to classify MRIs as either benign or affected by brain cancer.

Gurusamy and Subramaniam [24] compare several machine learning classifiers to determine the best one for discriminating between benign and malignant brain cancer MRIs. They evaluate Support Vector Machine, Neural Network, Naive Bayes, and k-nearest neighbors classification algorithms. The results show that the Support Vector Machine is the most effective model for detecting whether an MRI is related to brain cancer.

Rathi et al. [25] explore 60 features, including 22 shape, 5 intensity, and 33 texture features, for brain cancer detection. After selecting the best features using principal component analysis, their Support Vector Machine model achieves an accuracy of 0.98 in detecting malignant brain cancer in MRIs.

Vani et al. [26] exploit the Support Vector Machine to build a model for detecting cancerous or non-cancerous brain MRIs.

Georgiadis et al., in reference [27], propose a probabilistic neural network using 36 textural features to distinguish between metastatic and primary tumors, as well as between gliomas and meningiomas.

Zhang et al. [28] consider the application of the Support Vector Machine algorithm, and the model uses wavelet entropy and Hu moment invariants for feature extraction.

Abidin et al., in reference [29], investigate the effectiveness of machine learning models built with the AdaBoost classifier in predicting brain metastasis and glioblastoma multiforme brain cancers, achieving a classification performance of 0.71.

Fuzzy cognitive maps are utilizsd by Papageorgiou and colleagues [30] to build a model distinguishing between low and high-grade brain cancers. The model, based on 100 patients, achieves an accuracy of 0.92.

Sajjad et al. [31] consider the detection of brain cancers and distinguishing between grades I, II, III, and IV. A convolutional neural network, specifically the VGG-19 architecture, is employed for brain tumor grade classification, achieving an accuracy of 0.90.

Genetic algorithm and Support Vector Machine are combined by Kharrat et al. [32] with 44 discrete wavelet-based features. The principal component analysis identifies the best features: mean of contrast, mean of homogeneity, mean of sum average, mean of sum variance, and range of autocorrelation.

Barker et al. [33] propose a method to detect brain cancer cases, classifying them into two possible diagnoses: glioblastoma multiforme and lower-grade glioma, achieving an accuracy of 93.1.

Hsieh et al. [34] distinguish between glioblastomas and diffuse lower-grade gliomas using 14 textual features as input for a model built with the logistic regression algorithm.

Vu and colleagues [35] propose a method to detect the presence of MicroVascular Proliferation, which is symptomatic of a high-grade tumor in brain glioma, using histopathological images.

Meningioma brain tumor classification is investigated by David et al. [36] by considering features based on two matrices: one containing the whole cell’s boundary and the other containing the boundary of some cells. These features are used as input for a Support-Vector-Machine-based model.

Qurat et al., in reference [37], employ first- and second-order texture feature extraction for benign and malignant brain cancer detection using a Support Vector Machine model.

In reference [38], Cui and colleagues use machine learning techniques to detect high-grade and low-grade brain cancers. They achieve a prediction accuracy of 0.92 by evaluating MRIs from 50 patients.

In reference [39], Amin et al. explore the effectiveness of neural networks in detecting whether an MRI is associated with brain cancer. They achieve an average recognition rate of 78%, considering three types of brain cancer, using a dataset containing a total of 30 MRIs.

Badran et al. [40] utilise the Neural Network algorithm to label MRIs as either benign or malignant tumors. The application of the Canny edge detection algorithm results in an inaccuracy of 15–16%.

In the method proposed by Xuan et al. [41], features based on texture, symmetry, and intensity are extracted from brain MRIs. The authors employ the AdaBoost algorithm to build a model for classifying the MRIs as normal or abnormal, achieving an accuracy of 96.82%.

In reference [42], Ibrahim et al. consider Neural Networks to classify brain MRIs, yielding an accuracy of 96.33%.

Mohsen et al. [43] propose a deep-learning-based method. They use the Fuzzy C-means algorithm to segment the MRIs and extract features using discrete wavelet transform. The designed deep neural network comprises seven hidden layers and achieves a precision and recall of 0.97 in classifying normal and malignant brain MRIs.

In reference [44], Afshar et al. discuss a method for detecting brain cancer grades, utilising convolutional neural networks with seven hidden layers, achieving an accuracy of 0.86 in brain cancer grade detection.

Zia and colleagues [45] investigate the same problem by employing discrete wavelet transform for feature extraction, principal component analysis for feature selection, and support vector machine for the classification task.

Cheng et al. [46] consider intensity histogram and gray level co-occurrence matrix features for brain cancer grade detection, reaching an accuracy of 0.91.

For a comparison of the proposed method with respect to the state-of-the-art literature, in Table 1 [47] we provide a comparison of the state-of-the-art methods in brain cancer detection. In the patient column, the number of evaluated patients is indicated, while the accuracy column displays the corresponding performance achieved. The focus column identifies the final aim of each method, with benign/malign denoting methods aimed at discriminating between benign and malignant brain cancer, and L/H indicating methods focused on discriminating between low- and high-grade brain cancer. The localisation column highlights works related to brain cancer grade detection that utilise Grad-CAM for cancer localisation (we mark a paper with the ✓ whether localisation is considered and with ✗ whether it is not considered by authors). The last row in Table 1 provides details about the dataset and the accuracy attained using the proposed method.

With respect to the cited works, the main novelty of the proposed contribution is the adoption of explainability aimed at providing the rationale behind the model prediction, by employing the Grad-CAM algorithm to highlight the areas of interest.

## 3. The Method

As previously mentioned, the proposed method utilises supervised machine learning, specifically deep learning, with the adoption of Convolutional Neural Networks (CNNs). CNNs are a type of artificial neural network particularly suited for image classification tasks, making them applicable in the context of diagnosing brain cancer.

In this method, CNNs are trained using labelled datasets that include images of healthy brains and brains affected by cancer. The network learns from these examples and extracts meaningful features from the images to differentiate between healthy and cancerous brain patterns. The training process involves iteratively adjusting the network’s parameters to minimise the classification error and improve its accuracy in distinguishing between the two classes.

Once the CNN is trained, it can be used to classify new, unseen brain images into healthy or cancerous categories based on the learned patterns. The network analyses the input image using its learned filters and identifies relevant features to make a prediction.

**Figure 1:**
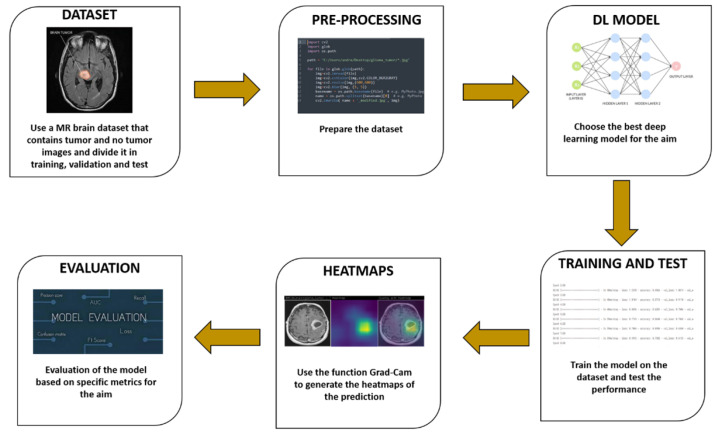
The proposed method for explainable brain cancer detection and localisation.

The workflow of the proposed method is shown in Figure 1.

The proposed method consists of six distinct steps:

1. The dataset used in machine learning methods, including those for brain cancer diagnosis, plays a crucial role in generating an effective model. It is essential to have a carefully labelled dataset that includes both healthy brain images and images of brains affected by cancer. To ensure the robustness and generalisability of the model, it is important to have a diverse and representative dataset. Medical specialists may use different imaging setups or protocols to capture brain images, so it is necessary to include images from various sources and imaging modalities. This variability helps the classifier learn patterns that are applicable across different scenarios, improving its ability to generalise and make accurate predictions on unseen data. By including a wide range of images, encompassing different patients, disease stages, imaging techniques, and variations in data acquisition, the trained model can better capture the complex and diverse nature of brain cancer. This increases the chances of obtaining a classifier that performs well on a variety of real-world scenarios and contributes to its clinical applicability. However, it is important to note that acquiring a diverse and representative dataset can be challenging due to factors such as data availability, privacy concerns, and ethical considerations. In summary, a well-constructed dataset with a good degree of variability is vital for training machine learning models for brain cancer diagnosis. It allows the model to learn from diverse examples and enhances its ability to generalise and make accurate predictions in real-world scenarios.
2. Once a dataset is obtained for brain cancer diagnosis, it is necessary to preprocess the images to ensure uniformity and remove any biases introduced by different imaging machines or settings. Preprocessing steps are essential for improving the consistency and quality of the dataset. One common preprocessing technique is to adjust the brightness of the images, particularly during the training phase. Randomly adjusting brightness can help mitigate any variations in image intensity caused by differences in imaging equipment or settings. By applying brightness adjustments, the model can learn to be less sensitive to these variations and focus more on the underlying features and patterns indicative of brain cancer. It is important to note that preprocessing techniques may vary depending on the specific requirements of the dataset and the characteristics of the images. Other preprocessing steps commonly used in medical imaging applications include image resizing, normalisation, noise reduction, and contrast enhancement. These techniques aim to standardise the input data and improve the effectiveness of the machine learning algorithms. The choice of preprocessing techniques should be based on a careful consideration of the dataset characteristics, the specific goals of the study, and domain knowledge from medical experts. It is also crucial to validate the impact of preprocessing steps on the model’s performance and ensure they do not introduce unintended biases or distortions in the data. In summary, the preprocessing of brain cancer images involves various techniques to standardise the dataset and remove the biases introduced by imaging equipment and settings. Randomly adjusting brightness is one such technique that helps improve consistency and reduce sensitivity to variations in image intensity. However, the selection and evaluation of preprocessing techniques should be done with care and consideration of the specific dataset and goals of the study.
3. Once the data collection and preprocessing phases are complete, the next step in developing a brain cancer diagnosis model is the selection of deep learning models. The literature offers a wide range of models to choose from, so the objective is to identify the most suitable one. Evaluating the accuracy of predictions is important, but it is also crucial to consider the quality of predictions and provide explainability. Explainability refers to understanding and interpreting the reasoning behind the model’s predictions. This is especially important in medical applications where decisions can have significant implications. Models that offer explainability can help medical professionals and researchers gain insights into the factors influencing the predictions and provide a clearer understanding of the decision-making process. In addition to selecting a suitable model, setting hyperparameters is another important step. Hyperparameters are values that determine the behavior and performance of the model during training. Examples of hyperparameters include the number of epochs (the number of times the model sees the entire dataset during training), batch size (the number of samples processed before updating the model’s parameters), and learning rate (the step size for adjusting the model’s parameters during training).

Selecting the appropriate hyperparameters requires careful consideration and experimentation. Different combinations of hyperparameters can significantly impact the model’s performance and convergence. Techniques such as grid search or random search can be used to explore different hyperparameter settings and identify the configuration that yields optimal results.

This paper explores various deep learning architectures based on convolutional neural networks, including VGG16, ResNet50, Alex_Net, and MobileNet. These architectures comprise essential layers as follows:

- *Conv2D*: This layer performs 2D convolution, such as spatial convolution over images. It generates a convolution kernel applied to the input layer, producing an output tensor.
- *MaxPooling2D*: This operation conducts maximum pooling on 2D spatial data. It downsamples the input across height and width, retaining the maximum value within each input window defined by the pool_size.
- *Flatten*: This layer transforms input into a flattened form, commonly transitioning from convolutional to fully connected layers. It does not affect batch size.
- *Dropout*: By applying Dropout, this layer randomly sets input units to 0 during training with a specified rate. This aids in preventing overfitting. Non-zero inputs are scaled by 1/(1 − rate), keeping their sum consistent.
- *Dense*: Neurons in this deep layer receive input from all previous layer neurons. It is widely used for classification tasks, involving matrix–vector multiplication with trainable parameters updated through back-propagation.

For comprehensive information on the models used, refer to the literatures [48,49,50,51].

After defining the models, training and testing ensue. A set of metrics, i.e., Accuracy, Precision, and Recall, gauge the prediction correctness. If the results are unsatisfactory, different hyper-parameter and model combinations are explored until the desired outcomes emerge.

The subsequent phase involves generating heatmaps using the Grad-CAM algorithm. This aims to offer visual explanations, showing not just prediction accuracy but also the retinal image areas responsible for classifications. Grad-CAM extracts gradients from model convolutional layers to convey graphical information during inference. These gradients capture high-level visual patterns, indicating influential regions in input images. As convolutional layers retain spatial data, Grad-CAM employs this to create heatmaps highlighting image regions driving model decisions. This provides a visual rationale for decisions made by the deep learning model. The Grad-CAM utilised in this study is based on the implementation introduced in the paper by Selvaraju et al. [8].

The Grad-CAM offers several advantages as a visualisation technique for understanding CNNs and their decision-making processes:

(a) Interpretability: Grad-CAM provides a transparent and interpretable way to visualise the CNN’s decision-making process. It allows researchers and practitioners to understand which parts of the input image were critical in influencing the network’s classification decision.
(b) No Architecture Modification: One significant advantage of Grad-CAM is that it does not require any changes or modifications to the CNN architecture. It can be applied to pre-trained models without the need for retraining, making it a convenient tool for visualising existing models.
(c) Localisation: Grad-CAM provides localisation information, indicating the exact regions within the input image that the network focused on while making its classification decision. This information is valuable for tasks where understanding what parts of the image contribute to the decision is crucial, such as medical image analysis or object detection.
(d) High-Level Visual Patterns: By using gradients from the final convolutional layers, Grad-CAM can capture high-level visual patterns in the input image. This makes it particularly useful for tasks that require understanding complex visual cues.
(e) Preservation of Spatial Information: Grad-CAM retains spatial information from the original input image, ensuring that the visualised heatmap aligns accurately with the relevant regions in the image.
(f) Applicability to Different Tasks: Grad-CAM is a versatile technique that can be applied to various CNN-based tasks, including image classification, object detection, and image segmentation. Its adaptability makes it a widely applicable tool in computer vision research.
(g) Model Debugging: When a CNN produces unexpected or erroneous results, Grad-CAM can be used as a debugging tool to visualise where the model focused its attention. This can help identify potential weaknesses or biases in the network’s decision-making process.
(h) Explainable AI: In contexts where explainability and transparency are essential, Grad-CAM can provide insights into how a CNN arrives at its predictions, increasing user trust and confidence in the model’s outputs.

Overall, Grad-CAM is a powerful tool that enhances our understanding of CNNs and helps bridge the gap between the “black-box” nature of deep learning models and the need for interpretability and transparency in AI systems.

4. The last step is the evaluation one, where we collect the metrics (i.e., Accuracy, Precision, and Recall) obtained from the testing of the employed models. Moreover, we consider the confusion matrix with the aim to understand the exact number of misclassifications per class.

## 4. Experimental Analysis

A dataset composed of 3000 brain medical images is gathered; in particular, 1500 images belong to patients affected by brain cancer and the remaining 1500 belong to healthy patients. The dataset we exploited (called *Br35H::Brain Tumor Detection 2020*, accessed on 20 August 2023) is freely available for research purposes on the Kaggle website (https://www.kaggle.com/datasets/ahmedhamada0/brain-tumor-detection, accessed on 20 August 2023).

**Figure 2:**
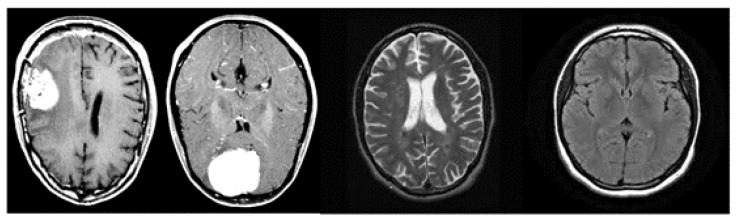
Examples of images belonging to the analysed dataset.

Figure 2 shows a set of images belonging to the analysed dataset; we can note that the images have a single angle (i.e., the one transversal to the height of the cortex).

The dataset, composed of a total of 3000 images, is subsequently split into 80/10/10 for the training, validation, and testing phases, respectively, thus 1800 (900 related to healthy patients and the remaining 900 to patients affected by brain cancer) images are considered for training, 600 (300 related to healthy patients and the remaining 300 to patients affected by brain cancer) for validation, and the remaining 600 (300 related to healthy patients and the remaining 300 to patients affected by brain cancer) for testing.

Then, we move to the deep learning model training. The following hyper-parameters are considered: 50 epochs, batch size equal to 32, 0.01 as learning rate, and an image size equal to 224 × 224 (with 3 channels). We have chosen these particular parameters in an empirical way, as from various tests carried out, we have found that the most satisfactory results have been obtained with these parameters.

As discussed in the previous section, we evaluate the effectiveness of the proposed method for brain cancer by computing the accuracy, precision, and recall metrics.

Table 2 shows the results of the experimental analysis.

As shown from the results in Table 2, the ResNet50 model seems to be the best one in the discrimination of cancerous and healthy brain medical images: as a matter of fact, it achieves an accuracy, a precision, and a recall equal to 99.67%. Also, the Alex_Net model achieves interesting performances, with accuracy, precision, and recall equal to 99.33%, significantly closer to the ones obtained using the ResNet50 model. The Alex_Net model obtains a value equal to 99.33% for the three metrics and the VGG16 achieves the lower values of the metrics, in comparison to the other models, with a value of 97.83%.

**Figure 3:**
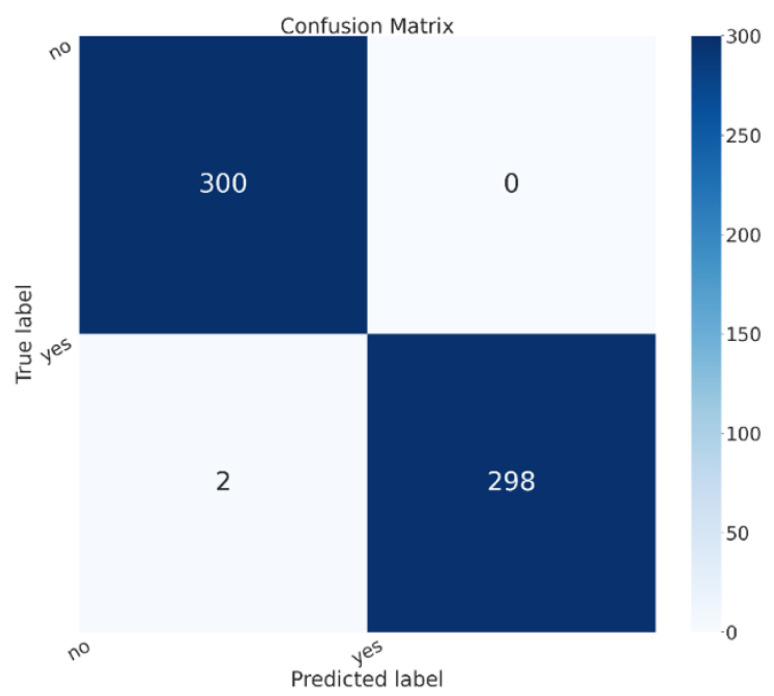
The confusion matrix for the ResNet50 model.

Figure 3 shows the confusion matrix for the model that obtained the best performances, i.e., the ResNet50 one.

In the confusion matrix shown in Figure 3, the yes label is related to brain cancer images, while no is related to healthy patients. In the confusion matrix, there are images shown related to the testing split of the dataset. We can see that 600 patients were considered in the testing phase, 300 belonging to the healthy class and 300 to the sick class; all 300 sick patients were correctly classified in the correct class (i.e., with the yes label in the confusion matrix in Figure 3), while 298 healthy patients were correctly classified as healthy (i.e., with the no label in the confusion matrix in Figure 3). Thus, only two healthy patients were classified as being affected by brain cancer, while 0 patients affected by brain cancer were incorrectly classified as healthy.

**Figure 4:**
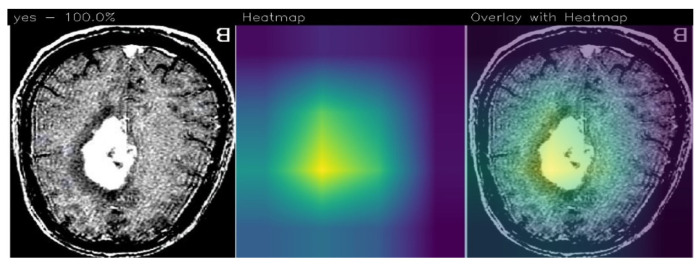
An example of prediction performed using the proposed method with the detail of tumor localisation from the ResNet50 model’s point of view.

In relation to explainability, in Figure 4 we show an example of localisation performed by the proposed method, in particular, there are areas (in yellow) where, from the proposed ResNet50 model’s point of view, there is disease localisation.

The GradCAM is an interpretability technique used to highlight areas in an image that contribute the most to a model’s prediction. It assigns different color intensities to these areas based on their importance from the model’s perspective.

In the context of brain tumor detection, GradCAM can help identify regions in the image that are particularly symptomatic of a brain tumor or are indicative of pathology. The technique assigns a yellow color to areas of extreme importance, which are highly influential in the model’s prediction and strongly associated with the presence of a brain tumor. These areas are considered particularly symptomatic.

Areas of minor importance, but still relevant to the pathology, are assigned a green color by GradCAM. These regions may contribute to the overall prediction but to a lesser degree compared to the yellow areas. They still provide valuable information related to the presence or characteristics of the pathology.

On the other hand, GradCAM uses a purple color to indicate areas that are not of interest for the detection of pathology. These regions are considered less important and do not significantly influence the model’s prediction.

By applying GradCAM, researchers and medical professionals can visualise and understand which areas of an image are driving the model’s decision-making process. This can provide insights into the features or patterns that the model considers indicative of a brain tumor or pathology, enhancing the interpretability and trustworthiness of the model’s predictions.

As we note, the Grad-CAM highlighted the areas related to brain cancer (i.e., the white ones), by showing the effectiveness of the proposed method in brain cancer detection and localisation. For this reason, we think that Grad-CAM may provide explainability behind the model’s prediction, as it highlights the area that the model judged to be symptomatic of the brain tumor.

One of the most interesting advantages deriving from the adoption of Grad-CAMs is the possibility of having the areas of the image responsible for a determined prediction (in this case, of brain cancer) starting from a model built for the classification task of images. The results relating to localisation do not appear in the paper, as the dataset provided contained only the image label and no localisation details. In any case, we submitted the images with the areas highlighted by the Grad-CAM to three expert radiologists belonging to different hospitals, and they confirmed that the Grad-CAM highlights the correct area of the pathology.

## 5. Conclusions and Future Work

Considering the diffusion of brain cancer, and the importance of an early diagnosis with the aim to start therapy as soon as possible, in this paper we proposed a method to discriminate between medical images related to healthy patients and patients affected by brain cancer. For this purpose, we exploit explainable deep learning, in particular, convolutional neural networks, and we adopt the Grad-CAM algorithm to highlight the areas symptomatic of brain cancer from the model’s point of view. Four different models are considered: VGG16, ResNet50, Alex_Net, and MobileNet. We evaluated the proposed method on 3000 brain images (1500 obtained from healthy patients and 1500 from patients affected by brain cancer), obtaining an accuracy equal to 99.67% with the ResNet50 model. During the testing phase, with the model obtaining the best performances (i.e., the ResNet50 one), a total of 600 patients were considered, with 300 categorised as healthy and 300 as sick. All 300 sick patients were correctly classified in the correct class, while 298 healthy patients were accurately identified as healthy. Only two healthy patients were misclassified as being affected by brain cancer, while no patients with brain cancer were incorrectly classified as healthy.

In future works, we plan to consider the detection of the cancer grade [47]. Moreover, we plan to form methods [52,53] with the aim of improving the obtained performances.

## Figures and Tables

**Table 1:** State-of-the-art comparison.

Reference	Patients	Accuracy	Focus	Localisation
[10]	n.a.	0.95	benign/malign	✗
[11]	121	0.91	benign/malign	✗
[9]	51	0.80	benign/malign	✗
[12]	130	0.98	benign/malign	✗
[13]	20	0.72	benign/malign	✗
[14]	n.a.	0.83	benign/malign	✗
[15]	17	0.97	benign/malign	✗
[16]	83	0.95	benign/malign	✗
[17]	45	0.89	benign/malign	✗
[18]	57	0.83	benign/malign	✗
[19]	130	0.91	L/H	✗
[20]	102	0.80	L/H	✗
[21]	101	0.99	benign/malign	✗
[22]	9	0.90	benign/malign	✗
[23]	70	0.98	benign/malign	✗
[24]	n.a.	0.97	benign/malign	✗
[25]	20	0.98	benign/malign	✗
[26]	130	0.82	L/H	✗
[27]	67	0.81	L/H	✗
[28]	n.a.	1	benign/malign	✗
[29]	52	0.71	L/H	✗
[30]	100	0.92	L/H	✗
[33]	302	0.93	L/H	✗
[34]	107	0.88	L/H	✗
[35]	190	0.94	L/H	✗
[36]	50	0.87	L/H	✗
[37]	n.a.	0.99	benign/malign	✗
[38]	50	0.92	benign/malign	✗
[39]	30	0.78	L/H	✗
[40]	n.a.	0.85	benign/malign	✗
[41]	140	0.96	benign/malign	✗
[42]	n.a.	0.96	benign/malign	✗
[43]	66	0.97	benign/malign	✗
Our method	3000	0.99	benign/malign	✓

**Table 2:** Experimental analysis results.

Metric	VGG16	Resnet50	Alex_Net	MobileNet
Accuracy	97.83%	99.67%	99.33%	98.5%
Precision	97.83%	99.67%	99.33%	98.5%
Recall	97.83%	99.67%	99.33%	98.5%
